# Relationship between serum alkaline phosphatase and poor 3-month prognosis in acute ischemic stroke patients with preserved renal function: results from Xi’an Stroke Registry Study of China

**DOI:** 10.1186/s12883-022-02779-y

**Published:** 2022-07-07

**Authors:** Zhongzhong Liu, Xuemei Lin, Lingxia Zeng, Qingli Lu, Pei Liu, Jing Wang, Yan Liu, Qiaoqiao Chang, Yan Wang, Chensheng Song, Fang Wang, Yaling Shi, Guozheng Liu, Qing Wang, Songdi Wu

**Affiliations:** 1 Department of Neurology, Xi’an No.1 Hospital, The First Affiliated Hospital of Northwest University, Xi’an, 710002 China; 2 Department of Epidemiology and Biostatistics, School of Public Health of Xi’an Jiaotong University Health Science Center, Xi’an, 710061 China

**Keywords:** Alkaline phosphatase, Risk factor, Acute ischemic stroke, Renal function, Prognosis

## Abstract

**Background:**

In recent years, alkaline phosphatase (ALP) has been considered as one of the independent risk factors of acute ischemic stroke (AIS) and leads to worse clinical outcomes in patients with renal failure. In this study, we aim to investigate whether serum ALP level is associated with poor early-term prognosis in relationship of AIS patients with preserved renal function.

**Methods:**

A prospectively collected database of AIS patients hospitalized in the Xi’an district of China from January to December, 2015 was analyzed. The demographics, serum ALP levels and stroke outcomes of all patients at 3 months were reviewed. Patients were routinely followed-up for 3 months. Serum ALP level was analyzed as a continuous variable and quintiles (Q1-Q5). Multivariate logistic regression model and a two-piecewise linear regression model were used to investigate the relationship and to determine the threshold effect regarding serum ALP levels and poor 3-month prognosis of AIS patients with preserved renal function.

**Results:**

Overall, 1922 AIS patients were enrolled with 62.3% of them being men. The risk of having a poor 3-month prognosis was significantly increased in Q1, Q2, Q3 and Q5, when compared to that in Q4 being as the reference. The highest risk was noted in Q5 (odds ratio 2.21, 95% confidence interval: 1.32–3.73, *P* = 0.003) after being adjusted for confounders. Further analysis revealed a J-shaped curvilinear relationship between ALP levels and a poor 3-month prognosis of strokes (optimal threshold ALP level = 90 U/L). The relationship between both parameters was not significantly affected by age, sex, drinking, hypertension and leukocyte count (stratified by 10 × 10^9^/L) (*P* for interaction > 0.05).

**Conclusions:**

Serum ALP was noted as an independent risk factor for a poor 3-month prognosis of AIS patients with preserved renal function. ALP levels higher than 90 U/L could cause an increased risk of a poor 3-month prognosis.

**Supplementary Information:**

The online version contains supplementary material available at 10.1186/s12883-022-02779-y.

## Introduction

Acute ischemic stroke (AIS) is a sudden neurologic dysfunction caused by focal brain ischemia, and is the leading cause of worldwide death as well as disability-adjusted life years in China [1, 2]. It is well known that the risks of AIS are various and mainly include atherosclerosis, hypertension, diabetes mellitus, hyperlipemia, smoking and hyperhomocysteinemia [3]. Clinical studies have demonstrated in recent years that serum alkaline phosphatase (ALP) might be one of the independent risk factors causing ischemic stroke, since high serum ALP levels would promote the vascular calcification through catalyzing the hydrolysis of organic pyrophosphate and subsequent atherosclerosis [4]. Furthermore, elevated ALP levels were also considered to be related with systemic inflammation, malnutrition and metabolic syndrome, which may result in worse clinical outcomes in patients with strokes [4].

Previous studies revealed that renal impairment is closely associated with adverse events of cerebrovascular diseases [5, 6]. Serum ALP levels could induce vascular calcification [7, 8], and mineral disorders are closely associated with cardiovascular and cerebrovascular disease-related mortality in patients with end-stage renal disease [7, 8]. However, few studies have so far examined the relationship between serum ALP levels and poor early-term prognosis in stroke patients without impaired renal function. Moreover, different countries (or even different regions of the same country) may present inconsistent results due to differences in economic levels, dietary habits and lifestyle. In order to provide a basis for ALP-based therapeutics in strokes, we investigated whether serum ALP level is associated with poor 3-month prognosis in AIS patients with preserved renal function in the Xi’an district of China in this study.

## Materials and methods

### Study population

**Fig. 1:**
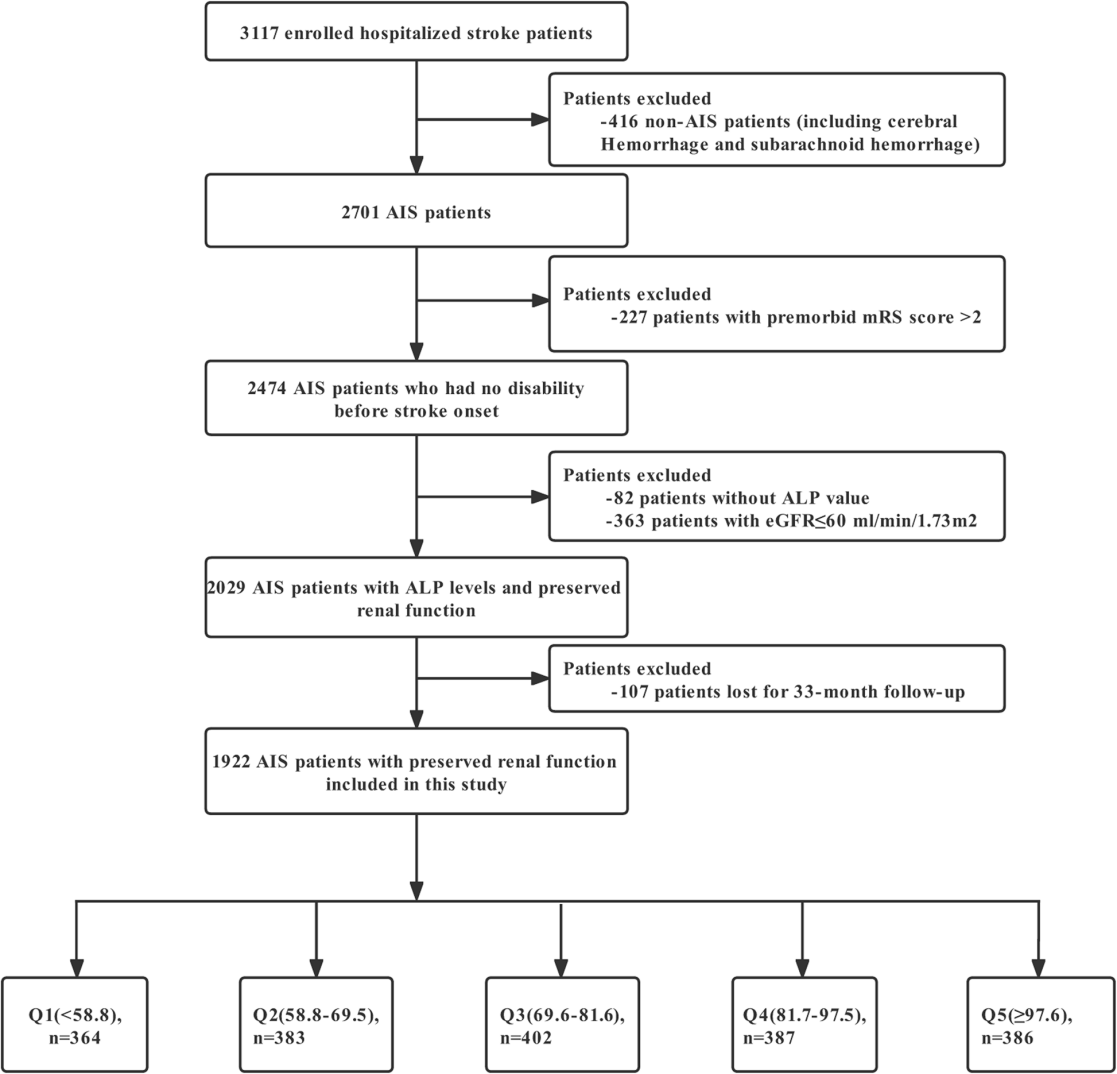
Flowchart of the screening and enrollment of the study participants. AIS, acute ischemic stroke; ALP, alkaline phosphatase; mRS, modified Rankin Scale; eGFR, estimated glomerular filtration rate; Q, quintiles

All stroke patients hospitalized at four tertiary grade A hospitals in the Xi’an district of China from January to December, 2015 were enrolled in this study. At the initial stage of the study, 3117 patients with strokes underwent integrated medical examinations and were followed up 1 and 3 months after the symptom onset. Among these patients, those without AIS (*n* = 416) and an ALP value (*n* = 82), and those with a premorbid modified Rankin Scale (mRS) score > 2 (*n* = 227) and an estimated glomerular filtration rate (eGFR) ≤ 60 mL/min/1.73 m^2^ (*n* = 363) were excluded. In addition, 107 patients lost for follow-up at 3 months were also excluded. Therefore, a total of 1922 AIS patients with preserved renal function were included in the final analysis. The screening process for enrolled patients are listed in Fig. 1. Consistent diagnostic criteria were used at all participating hospitals. Patient enrollment started in January of 2015 and follow-up was completed in February of 2017.

### Standard protocol approvals and patient consents

This study was conducted in accordance with the guiding principles of the Declaration of Helsinki and was approved by the academic committee of Xi’an No. 1 Hospital and the ethics committee of each participating hospital [Approval No. 2014(5)]. All patients provided written and oral informed consents.

### Baseline data collection

This multicenter observational cohort study used data from the Stroke Data Bank of Xi’an [9]. We collected baseline data including medical history, assessments at admission and laboratory test results (Table 1). The relevant risk factors, definitions and criteria for the medical history were the same as those in the Chinese Intracranial Atherosclerosis study [10]. The ALP level was analyzed and was treated as both a continuous and categorical variable (quintiles Q1-Q5). The ranges of quintiles (Q1-Q5) were as follows: Q1, < 58.8 U/L; Q2, 58.8–69.5 U/L; Q3, 69.6–81.6 U/L; Q4, 81.7–97.5 U/L, and Q5, ≥ 97.6 U/L. The eGFR was calculated using the Chronic Kidney Disease Epidemiology Collaboration creatinine equation with an adjusted coefficient of 1.1 for the Asian population [11, 12].

**Table 1:** Baseline and biochemical characteristics by ALP quintiles (Q1-Q5) in patients with AIS with preserved renal function

Variables	Overall (*n* = 1922)	ALP Quintiles, U/L					*P* value
		Q1 (*n* = 364)	Q2 (*n* = 383)	Q3 (*n* = 402)	Q4 (*n* = 387)	Q5 (*n* = 386)	
Age, years	63.1 ± 12.0	63.5 ± 12.6	62.8 ± 12.1	63.0 ± 11.8	62.8 ± 12.5	63.4 ± 11.0	0.879
Sex, n (%)							0.002
man	1198(62.3)	241(66.2)	260(67.9)	241(60)	243(62.8)	213(55.2)	
women	724(37.7)	123(33.8)	123(32.1)	161(40)	144(37.2)	173(44.8)	
Educational level, n (%)							0.336
Elementary or below	888(46.2)	155(42.6)	177(46.2)	200(49.8)	170(43.9)	186(48.2)	
Middle school	386(20.1)	75(20.6)	85(22.2)	66(16.4)	81(20.9)	79(20.5)	
High school or above	648(33.7)	134(36.8)	121(31.6)	136(33.8)	136(35.1)	121(31.3)	
Smoking, n (%)							0.003
Never smoking	1057(55.0)	204(56)	204(53.3)	232(57.7)	194(50.1)	223(57.8)	
Smoking cessation	375(19.5)	88(24.2)	75(19.6)	72(17.9)	87(22.5)	53(13.7)	
Current smoking	490(25.5)	72(19.8)	104(27.2)	98(24.4)	106(27.4)	110(28.5)	
Drinking, n (%)	473(24.6)	84(23.1)	108(28.2)	96(23.9)	103(26.6)	82(21.2)	0.169
Prior stroke, n (%)	504(26.2)	102(28)	103(26.9)	110(27.4)	95(24.5)	94(24.4)	0.694
NIHSS score on admission, n (%)	3.0(1.0,5.0)	3.0(1.0,5.0)	3.0(1.0,5.0)	3.0(1.0,5.0)	4.0(1.0,5.0)	4.0(1.0,6.0)	0.059
Pneumonia during hospitalization, n (%)	67(3.5)	21(5.8)	7(1.8)	4(1)	21(5.4)	14(3.6)	0.001
BMI, kg/m^2^	24.0 ± 3.5	24.1 ± 3.9	24.1 ± 3.7	24.0 ± 3.4	23.9 ± 3.0	23.8 ± 3.3	0.824
SBP on admission, mmHg	144.7 ± 21.0	142.9 ± 20.2	143.5 ± 20.5	144.9 ± 20.3	146.0 ± 21.4	145.9 ± 22.5	0.158
DBP on admission, mmHg	85.7 ± 12.2	85.2 ± 11.0	84.0 ± 12.4	86.1 ± 12.0	86.5 ± 12.4	86.5 ± 12.7	0.019
Hypertension, n (%)	1313(68.3)	247(67.9)	244(63.7)	281(69.9)	274(70.8)	267(69.2)	0.24
Diabetes mellitus, n (%)	409(21.3)	68(18.7)	87(22.7)	87(21.6)	72(18.6)	95(24.6)	0.186
Atrial fibrillation, n (%)	106(5.5)	20(5.5)	24(6.3)	16(4)	22(5.7)	24(6.2)	0.623
**Laboratory findings**							
Total cholesterol, mmol/L	4.4 ± 1.0	4.3 ± 1.0	4.4 ± 1.0	4.4 ± 1.1	4.4 ± 1.0	4.4 ± 1.0	0.252
Triglycerides, mmol/L	1.7 ± 1.3	1.5 ± 1.1	1.7 ± 1.3	1.7 ± 1.4	1.8 ± 1.4	1.7 ± 1.2	0.177
HDL-C, mmol/L	1.1 ± 0.3	1.1 ± 0.3	1.1 ± 0.3	1.1 ± 0.3	1.1 ± 0.3	1.1 ± 0.3	0.9
LDL-C, mmol/L	2.6 ± 0.8	2.5 ± 0.8	2.6 ± 0.8	2.6 ± 0.9	2.6 ± 0.8	2.6 ± 0.9	0.356
Fasting blood glucose, mmol/L	5.9 ± 2.3	5.5 ± 1.7	5.9 ± 2.3	5.8 ± 2.2	6.0 ± 2.5	6.2 ± 2.7	0.004
ALT, U/L	19.0(14.0,28.0)	17.2(13.0,26.0)	18.0(14.0,26.0)	19.0(14.0,29.0)	21.0(14.3,30.0)	21.0(15.1,29.8)	< 0.001
AST, U/L	21.0(17.0,27.1)	20.0(16.1,27.0)	20.0(17.0,25.0)	22.0(17.0,27.0)	22.0(17.0,30.0)	22.0(18.0,29.4)	< 0.001
ALP,U/L	79.3 ± 27.8	48.3 ± 10.0	64.1 ± 3.0	75.4 ± 3.4	88.5 ± 4.4	118.3 ± 30.7	< 0.001
Homocysteine, μmol/L	21.6 ± 14.3	19.4 ± 12.7	21.5 ± 14.9	20.7 ± 13.3	23.1 ± 16.5	23.1 ± 13.9	0.015
Serum creatinine, mg/dL	0.8 ± 0.2	0.8 ± 0.2	0.8 ± 0.2	0.8 ± 0.2	0.8 ± 0.2	0.8 ± 0.2	0.038
eGFR, mL/min/1.73 m^2^	80.6 ± 10.6	81.2 ± 10.2	80.7 ± 11.0	80.8 ± 10.9	80.2 ± 10.5	80.1 ± 10.3	0.613
Blood Urea Nitrogen, mmol/L	4.9 ± 1.6	4.9 ± 1.6	4.9 ± 1.5	4.8 ± 1.5	5.1 ± 1.8	4.9 ± 1.4	0.221
Uric Acid, μmol/L	285.2 ± 91.1	280.6 ± 92.6	291.4 ± 92.7	285.0 ± 88.9	292.3 ± 94.2	276.7 ± 86.5	0.08
Leukocyte values, × 10^9^/L	6.9 ± 2.4	6.5 ± 2.2	6.7 ± 2.2	6.7 ± 2.2	7.1 ± 2.6	7.2 ± 2.5	< 0.001

*Abbreviations: NIHSS* National Institutes of Health Stroke Scale, *HDL-C* High density lipoprotein cholesterol, *LDL-C* Low density lipoprotein cholesterol, *ALT* Alanine aminotransferase, *AST* Aspartate aminotransferase, *ALP* Alkaline phosphatase, *eGFR* estimated glomerular filtration rate, *BMI* Body mass index, *SBP* Systolic blood pressure, *DBP* Diastolic blood pressure

Q1: < 58.8 U/L, Q2: 58.8–69.5 U/L, Q3: 69.6–81.6 U/L, Q4: 81.7–97.5 U/L, Q5: ≥ 97.6 U/L

### Clinical outcome assessments

AIS Patients with preserved renal function were defined as those with an eGFR > 60 mL/min/1.73 m^2^. The observed endpoint event in this study was a poor 3-month prognosis which was defined as a mRS score of 3–6 at 3-month follow-up (score ranges from 0 [no symptoms] to 6 [death]) [13].

### Serum ALP measurements

Fasting venous blood samples were collected within 24 h of admission and serum ALP levels of unfrozen samples were detected by automatic enzymatic method at each participating hospital. ALP was measured in accordance with internationally recommended standards [14].

### Follow-up

All patients were followed up 1 and 3 months after AIS diagnosis. The follow-up time error was not longer than 5 days. All enrolled patients were followed up by trained study coordinators through telephone calls or face-to-face interviews. For those patients with a poor prognosis, the dates of events were recorded. Patients who refused to continue in this study or who could not be contacted by telephone after three attempts per day for five consecutive working days were considered lost for follow-up.

### Statistical analyses

Continuous variables are presented as Mean ± SD and categorical variables as percentages. One-way ANOVA was used to assess differences between groups for normally distributed continuous variables; the chi-square test and trend test were used for categorical variables. When the sample did not satisfy the normal distributions, the Mann–Whitney U-test was used. Multiple groups were compared using the Kruskal–Wallis rank-sum test. Fisher’s exact test was used when the theoretical frequency was < 10. Univariate and multivariable logistic regression models were used to calculate odds ratios (OR) and 95% confidence intervals (CI) for the relationship between ALP level and a poor 3-month prognosis. The covariates were included as potential confounders in model II if they changed the estimates of ALP levels by > 10% with respect to a poor 3-month prognosis or if they were significantly associated with a poor 3-month prognosis. A generalized additive model was used to evaluate the nonlinear relationship between ALP level and a poor 3-month prognosis in AIS patients with preserved renal function. In plotting the relationship between ORs and ALP levels, the group with the lowest effect value was considered as the reference group. Based on the logistic curve fitting, a two-piecewise linear regression model was used to identify the threshold effect. Subgroup analyses were conducted for age, sex, drinking and hypertension using stratified logistic regression models. The interaction among subgroups was tested using a likelihood ratio test. A two-tailed *p* value of < 0.05 was considered statistically significant. All analyses were performed with R statistical software version 3.3.2 (http://www.R-project.org; The R Foundation) and EmpowerStats (http://www.empowerstats.com; X&Y Solutions, Inc., Boston, MA, USA).

## Results

### Baseline characteristics

A total of 1922 eligible subjects (1198 men, 724 women) were enrolled in this study. The mean age was 63.1 ± 12.0 years and the mean ALP level was 79.3 ± 27.8 U/L. The baseline demographic, clinical and biochemical characteristics of the ALP quintiles (Q1-Q5) were compared (Table 1). Higher ALP levels were more likely to occur in women, current smokers as well as those with pneumonia during hospitalization and high diastolic blood pressure (DBP) on admission. Furthermore, the ALP level was directly proportional to fasting blood glucose, alanine aminotransferase (ALT), aspartate aminotransferase (AST), homocysteine, serum creatinine and leukocyte values. The ALP quintiles had no significant differences in age, educational level, drinking habits, prior stroke, National Institutes of Health stroke scale (NIHSS) scores on admission, body mass index (BMI), systolic blood pressure (SBP) on admission, hypertension, diabetes mellitus, atrial fibrillation, total cholesterol, triglycerides, HDL-C, LDL-C, eGFR, blood urea nitrogen or uric acid levels.

### The relationship between serum ALP levels and a poor 3-month prognosis in AIS patients with preserved renal function

Table 2 shows the results of the crude and multivariable-adjusted logistic regression models (Models I and II). First, an analysis was conducted using the ALP level as the continuous variable. For every 10-unit increase (10 U/L) in ALP, the risk of a poor 3-month prognosis increased by 6% in the crude model and model I (crude model and Model I: OR 1.06, 95% CI 1.02–1.11, *P* = 0.007). After being adjusted for potential confounding factors, the risk of having a poor 3-month prognosis increased by 4% in model II (OR 1.04, 95% CI 1.01–1.09, *P* = 0.041). Thereafter, ALP levels were analyzed as categorical variables (quintiles Q1-Q5). After adjusting for potential confounding factors, patients in Q1, Q2, Q3 and Q5 had a higher risk of having a poor 3-month prognosis than those in Q4 being as the reference quintile. The highest risk was noted in Q5 (OR 2.21, 95% CI 1.32–3.73, *P* = 0.003; Table 2). Although there was a significant difference in each group when compared with Q4, the trend test analysis showed no significant difference in the trend of increased risk in each quintile of model II (*P* for trend = 0.762). In a subgroup analysis, the relationship between ALP levels (Q1-Q5) and a poor 3-month prognosis was not significantly altered by age, sex, drinking, hypertension and leukocyte count (stratified by 10 × 10^9^/L); *P* for interaction > 0.05; Table 3).

**Table 2:** Multivariate analysis of the relationship between ALP levels and poor 3-month prognosis in AIS patients with preserved renal function

Variable	Crude OR(95%CI)	*P* value	Model I OR(95%CI)	*P* value	Model II OR(95%CI)	*P* value
ALP, per 10 unit increase	1.06(1.02 ~ 1.11)	0.003	1.06(1.02 ~ 1.11)	0.007	1.04(1.01 ~ 1.09)	0.041
ALP quintile						
Q1	1.27(0.82 ~ 1.97)	0.276	1.26 (0.81 ~ 1.96)	0.313	1.84(1.07 ~ 3.16)	0.028
Q2	1.17(0.76 ~ 1.82)	0.472	1.21 (0.78 ~ 1.89)	0.396	2.11(1.23 ~ 3.63)	0.007
Q3	1.32(0.87 ~ 2.02)	0.196	1.33 (0.86 ~ 2.05)	0.197	2.13(1.25 ~ 3.60)	0.005
Q4	Reference	-	Reference	-	Reference	
Q5	1.80(1.2 ~ 2.71)	0.005	1.81 (1.19 ~ 2.75)	0.005	2.21(1.32 ~ 3.73)	0.003
Trend test		0.171		0.183		0.762

*Notes:* Crude adjust none; Model I adjusted for age, sex; Model II adjusted for model I plus smoking, drinking, prior stroke, pneumonia during hospitalization, NIHSS score at admission, BMI, serum creatinine, eGFR; hypertension, atrial fibrillation, diabetes mellitus, ALT and leukocyte count

*Abbreviations: ALP* Alkaline phosphatase, *eGFR* estimated glomerular filtration rate, *OR* Odds ratios, *CI* Confidence interval, *Q* Quintile

Q1: < 58.8 U/L, Q2: 58.8–69.5 U/L, Q3: 69.6–81.6 U/L, Q4: 81.7–97.5 U/L, Q5: ≥ 97.6 U/L

**Table 3:** Subgroup analyses of the relationship between ALP quintiles (Q1-Q5) and poor 3-month prognosis in AIS patients with preserved renal function

Confounding factor category	ALP level quintiles(Q1-Q5)					P for trend	P for interaction
	Q1	Q2	Q3	Q4	Q5		
Age, years							0.188
< 65	2.28(0.69 ~ 7.52)	3.39(1.03 ~ 11.1)	5.33(1.76 ~ 16.16)	Reference	4.78(1.59 ~ 14.33)	0.434	
≥ 65	1.34(0.75 ~ 2.42)	1.41(0.78 ~ 2.56)	1.29(0.71 ~ 2.36)	Reference	1.43(0.79 ~ 2.61)	0.721	
Sex							0.243
man	1.83(0.89 ~ 3.76)	2.39(1.17 ~ 4.87)	2.83(1.39 ~ 5.77)	Reference	2.24(1.07 ~ 4.67)	0.620	
women	1.49(0.68 ~ 3.27)	1.24(0.55 ~ 2.79)	1.03(0.48 ~ 2.18)	Reference	1.63(0.81 ~ 3.29)	0.835	
Drinking							0.851
yes	1.55(0.88 ~ 2.71)	1.8(1.02 ~ 3.18)	1.76(1.01 ~ 3.07)	Reference	1.97(1.15 ~ 3.38)	0.979	
no	1.19(0.32 ~ 4.42)	1.37(0.39 ~ 4.86)	1.94(0.56 ~ 6.68)	Reference	1.05(0.26 ~ 4.32)	0.699	
Hypertension							0.647
yes	1.86(0.72 ~ 4.82)	1.75(0.68 ~ 4.55)	1.62(0.62 ~ 4.24)	Reference	1.87(0.72 ~ 4.86)	0.646	
no	1.29(0.69 ~ 2.4)	1.55(0.83 ~ 2.89)	1.82(1 ~ 3.29)	Reference	1.78(0.99 ~ 3.22)	0.696	
Leukocyte count, × 10^9^/L							0.720
< 10	1.71(0.98 ~ 2.99)	1.9(1.08 ~ 3.35)	1.94(1.12 ~ 3.37)	Reference	1.99(1.14 ~ 3.45)	0.683	
≥ 10	0.91(0.14 ~ 5.82)	1.13(0.2 ~ 6.44)	2.04(0.34 ~ 12.03)	Reference	2.24(0.52 ~ 9.66)	0.455	

*Notes:* adjusted for smoking, prior stroke, NIHSS score at admission, BMI, serum creatinine, eGFR; Pneumonia during hospitalization, atrial fibrillation, diabetes mellitus, ALT,

*Abbreviations: ALP* Alkaline phosphatase, *eGFR* estimated glomerular filtration rate, *OR* Odds ratios, *CI* Confidence interval, *Q* Quintiles

Q1: < 58.8 U/L, Q2: 58.8–69.5 U/L, Q3: 69.6–81.6 U/L, Q4: 81.7–97.5 U/L, Q5: ≥ 97.6 U/L

### Threshold effect analysis of ALP levels regarding a poor 3-month prognosis in AIS patients with preserved renal function

**Fig. 2:**
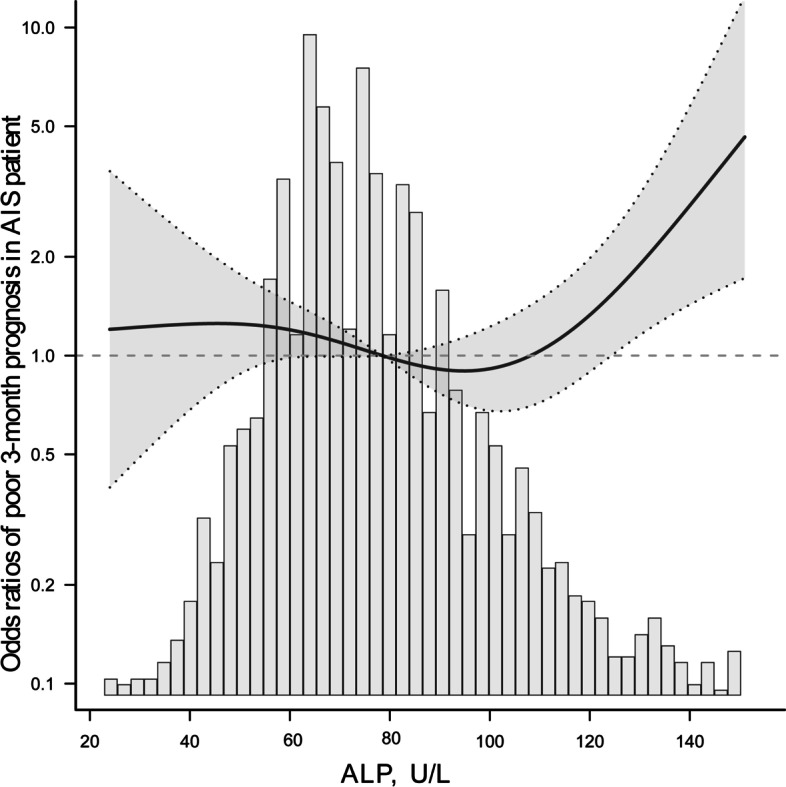
The fitted curve showing the relationship between serum ALP level and poor 3-month prognosis in AIS patients with preserved renal function. The solid and dashed lines indicate adjusted odds ratios and the 95% confidence interval bands, respectively. Histogram represents the kernel density distribution of ALP. ALP, alkaline phosphatase

After being adjusted for potential confounding factors, smoothing curve analysis showed a J-shaped-curve relationship between ALP levels and a poor 3-month prognosis in AIS patients with preserved renal function (Fig. 2). The two-piecewise linear regression model analysis showed that the optimal threshold value of ALP levels was 90 U/L. To the left of the threshold value, the risk of a poor 3-month prognosis was not significantly associated with ALP levels (per 10 U/L increase) until it was up to 90 U/L (OR 0.99, 95% CI 0.87–1.12, *P* = 0.857); whereas to the right of the optimal threshold value, the risk of a poor 3-month prognosis significantly increased (OR 1.57, 95% CI 1.27–1.93, *P* < 0.001; Table 4). The likelihood ratio test indicated that there was a significant difference when two different linear regression models (*P* = 0.001) were compared.

**Table 4:** Threshold effect analysis of ALP levels and poor 3-month prognosis in patients with AIS with preserved renal function

Outcome	OR(95%CI)	*P* value
One-line linear regression model, per 10 unit increase	1.04(1.01,1.09)	0.041
Two-piecewise linear regression mode		
ALP level < 90 U/L, per 10 unit increase	0.99 (0.87,1.12)	0.857
ALP level ≥ 90U/L, per 10 unit increase	1.57(1.27,1.93)	< 0.001
Likelihood Ratio test	0.001	

*Notes:* adjusted variables included age, sex, smoking, drinking, prior stroke, NIHSS score at admission, pneumonia during hospitalization, BMI, serum creatinine, eGFR; hypertension, atrial fibrillation, diabetes mellitus, ALT, leukocyte count

*Abbreviations: ALP* Alkaline phosphatase, *eGFR* estimated glomerular filtration rate, *OR* Odds ratios, *CI* Confidence interval, *Q* Quintile

## Discussion

In this multicenter perspective cohort study, we found that an elevated serum ALP level was an independent risk factor for a poor 3-month prognosis in AIS patients with preserved renal function in the Xi’an district of China. Such relationship was not significantly affected by age, sex, drinking, hypertension and leukocyte count (stratified by 10 × 10^9^/L). We also found that the relationship between ALP levels and a 3-month poor prognosis appeared as a J-shaped curve and the optimal threshold value was 90 U/L.

Previous studies showed that ALP levels could serve as a biomarker for the diagnosis and prognosis of stroke. Moreover, elevated serum ALP levels appeared to be associated with stroke severity, increased vascular deaths and recurrent vascular events, and an increased incidence of early mortality [15–17]. The effect of ALP on prognosis in cardiovascular diseases has recently attracted increasing attention, and ALP level is associated with vascular calcification. Vascular calcification often leads to increased vascular sclerosis and decreased vascular compliance being closely related to atherosclerosis [18, 19].

In addition, elevated serum ALP levels were associated with the risk of cardiovascular morbidity and mortality in patients with renal failure [7, 8, 20]. However, as most previous studies pooled together stroke patients with and without renal failure, their results could not rule out the effect of abnormal renal function, even with adjustments for confounding factors. Although some studies recently focused on this issue, few of them have explored the relationship between serum ALP levels and a poor 3-month prognosis in AIS patients with preserved renal function.

In this study, the main patient variables were comparable between patients lost and not lost for follow-up at the end of 3 months (Supplementary table 1). Overall, the data showed a random pattern regarding the patients who were lost for follow-up in this study. Thus, the results of this study were not markedly affected by the loss for follow-up. The analyzed population was a good representation of the patients enrolled in this study. On the basis of ensuring sample representativeness, we attempted to explore the relationship between serum ALP levels and a poor 3-month prognosis in AIS patients with preserved renal function. Our findings showed that 5% of the patients had a poor prognosis at the end of the 3-month follow-up period. The analysis of multivariate-adjusted logistic regression showed that for every 10-U/L increase in the ALP level, the risk of a poor 3-month prognosis increased by 4%. The patients in the Q5 group had a 1.21-fold risk of a poor 3-month prognosis in comparison with those in Q4 group. These results imply that an elevated serum ALP level is an independent risk factor for a poor 3-month prognosis in AIS patients with preserved renal function. Clinicians should be concerned about rapidly elevated serum ALP levels, as such an increase may be associated with an even higher risk of a poor 3-month prognosis.

The relationship and optimal range of serum ALP levels regarding a poor 3-month prognosis were not clearly elucidated in previous studies. To clarify this issue, the threshold effect and dose–response relationship between ALP levels and a poor 3-month prognosis were systematically analyzed in this large-scale cohort study, and the results confirmed a nonlinear relationship. After being adjusted for potential confounders, the risk of a poor 3-month prognosis was significantly increased in Q1, Q2, Q3 and Q5 (with the highest risk in Q5) when compared to that in Q4 being considered as the reference group. Logistic curve fitting was performed using the ALP level as a continuous variable, and it showed a J-shaped-curve relationship between ALP levels and a poor 3-month prognosis (Fig. 2). The optimal ALP range was observed in Q4, and the optimal threshold value was 90 U/L (Table 4). Thus, values above 90 U/L may lead to an increase in the risk of a poor 3-month prognosis in this population.

A previous long-term study reported that the risk of all-cause mortality was 1.78-fold higher in the highest quintile of ALP (≥ 97 U/L) than in the lowest quintile (< 57 U/L). The curve fitting results showed that the relationship between ALP levels and all-cause mortality was close to a positive correlation straight line [21]. The Chinese National Stroke Registry Study showed that patients with their ALP levels being > 98 U/L had a 36% higher risk of all-cause mortality in a 1-year follow-up period, compared to those with ALP levels < 59 U/L. The curve fitting results also showed a linear relationship [22].

In this study, the poor 3-month prognosis of stroke caused by increased ALP level was similar to the previous studies, but low ALP level also suggested that the increased risk of 3-month prognosis of stroke patients was inconsistent with those in the previous results. This inconsistency may be due to different populations and durations of follow-up in various studies. We conducted this study with 3-month follow-up and excluded patients with eGFR ≤ 60 mL/min/1.73 m^2^. In addition, differences in the study setting may have led to the inconsistent results because the patients in the present study were from the Xi’an district, which corresponds to the lower valley of the Wei River in the Central Shaanxi Plain of northwestern China. Apart from the different clinical characteristics from our included population, some previous basic investigations showed that low ALP levels could increase the amount of inhibitory pyrophosphate resulting in abnormal metabolism of pyridoxine-5 '-phosphate (the main form of vitamin B6) and hypomineralization of the skeleton. The loss of mineralization could cause a variety of symptoms including softening of bone, bowing and spontaneous breakage of bones, rickets and tooth defects [23, 24]. However, whether the reduced ALP level could be associated with poor 3-month prognosis in AIS patient with preserved renal function needs to be further explored and verified by subsequent relevant basic experiments. In addition, unlike those previous studies that included covariates based on statistical significance or clinical experience, the covariates were included as potential confounders in our study if they changed the estimates of ALP levels by > 10% with respect to a poor 3-month prognosis or if they were significantly associated with a poor 3-month prognosis. This may also be one of the reasons why the results are inconsistent with the previous studies. Our results suggested that clinicians could evaluate the probability of a poor 3-month prognosis according to the serum ALP levels at admission to strengthen the awareness of standardized treatment and complications and to reduce the risk of a poor early-term prognosis in AIS patients with preserved renal function in this district.

Previous studies have shown that ALP levels are associated with many risk factors for stroke, including age, sex, drinking [25], hypertension [26] and inflammation [23]. To further analyze the possible effects of these factors, the subgroup analysis in this study showed that the relationship between ALP levels (Q1-Q5) and a poor 3-month prognosis was not significantly altered by age, sex, drinking, hypertension and leukocyte count (stratified by 10 × 10^9^/L). These results suggested a relatively stable relationship and they provided a theoretical basis for clinicians to institute appropriate preventive managements and therapeutics for these patients.

The mechanisms underlying the relationship between elevated serum ALP levels and a poor 3-month prognosis are not fully known. There are several probable explanations to clarify the relationship. First, serum ALP may affect the permeability of the blood–brain barrier. ALP may also play an important role in the maintenance and integrity of the blood–brain barrier and in the transport of proteins across the barrier. An elevated serum ALP level may cause dysfunction in the transport of these proteins, leading to the breakdown of the blood–brain barrier and death of neurons [27–29]. Second, ALP may be associated with neuroinflammation. An elevated serum ALP level is highly characteristic of an inflammatory response in many diseases. Previous studies have shown that ALP plays a critical role in suppressing neuroinflammation by interfering with immune activation and responses [30]. Infections of the respiratory and urinary tracts are very common after stroke [31, 32]. These may be associated with infection-induced inflammation and also be involved in ALP upregulation in patients with stroke. Third, ALP can alter vascular homeostasis. Previous studies have speculated that serum ALP is associated with vascular calcification and microcirculation disorders, causing the hardening of vascular walls, myocardial ischemia and further cerebrovascular dysfunction and cerebral ischemia [33, 34]. In addition, our study showed that the relationship between serum ALP levels and a poor 3-month prognosis followed a threshold effect with an increased risk at ALP levels > 90 U/L, especially in Q5, indicating that a rapid elevation in ALP levels may have a markedly deleterious effect and result in a poor prognosis. This also implies that an abnormal serum ALP level is a potential treatment target to improve poor early-term prognosis. However, this notion needs to be verified in subsequent experiments.

Our study has some limitations. First, the four selected hospitals were not randomly chosen for this study. All the selected hospitals were tertiary grade A but may not represent the status quo of AIS patients with preserved renal function at other community hospitals. Second, this study mainly focused on the relationship between serum ALP levels at admission and a poor 3-month prognosis, and failed to timely collect the stress level of ALP at symptom onset and the ALP level after discharge. In addition, the timepoint of each patient was not recorded at measurement. These deficiencies may have an impact on the results. Third, the TOAST (Trial of Org 10,172 in acute stroke treatment) classification of stroke was not included in our study data, so the different types of stroke were not analyzed. Fourth, Inflammation-related indicators such as C-reactive proteins and hypersensitive C-reactive proteins were not included in our data, that may result in the failure to fully consider the inflammatory response of patients in the analysis. Finally, possible complications such as biliary tract disease and liver disease may also affect serum ALP levels. The lack of data for these indicators is another limitation of our study.

## Conclusions

In summary, this study indicates that increased serum ALP levels are an independent risk factor for a poor 3-month prognosis in AIS patients with preserved renal function, and the relationship between these two parameters revealed a J-shaped curve with an optimal threshold value of 90 U/L. Thus, ALP values higher than 90 U/L are associated with an increased risk of a poor 3-month prognosis in AIS patients with preserved renal function. Our findings should help physicians to assess the risk for a poor 3-month prognosis among this population and to provide an appropriate ALP range to guide clinical practice.

## Supplementary Information

**Additional file 1:** **Supplementary table 1.** Differences in clinical characteristics of the study groups were compared with those of the lost follow-up groups.

## Data Availability

The data that support the findings of this study are available on request from the corresponding author. The data are not publicly available due to privacy or ethical restrictions.

## Abbreviations

ALP: Alkaline phosphatase
ALT: Alanine aminotransferase
AST: Aspartate aminotransferase
AIS: Acute ischemic stroke
BMI: Body mass index
CIs: Confidence intervals
DBP: Diastolic blood pressure
eGFR: Estimated glomerular filtration rate
HDL-C: High-density lipoprotein cholesterol
LDL-C: Low-density lipoprotein cholesterol
mRS: Modified Rankin Scale; NIHSS: National Institutes of Health stroke scale
ORs: Odds ratios
Q: Quintile
SD: Standard deviation
SBP: Systolic blood pressure
TOAST: Trial of Org 10,172 in acute stroke treatment
