# Supplementary material for: Relationship between serum alkaline phosphatase and poor 3-month prognosis in acute ischemic stroke patients with preserved renal function: results from Xi’an Stroke Registry Study of China
Source: BMC Neurol. 2022 Jul 7;22:249. doi: 10.1186/s12883-022-02779-y (PMC9260988; doi:10.1186/s12883-022-02779-y)
Supplement: Supplementary file 1 — Additional file 1: Supplementary table 1. Differences in clinical characteristics of the study groups were compared with those of the lost follow-up groups. [file 12883_2022_2779_MOESM1_ESM.docx]

Supplementary table 1 Differences in clinical characteristics of the study groups were compared with those of the lost follow-up groups

| Variables | Overall  (n=2029) | Not loss to follow-up  at 3 month  (n=1922) | Loss to follow-up  at 3 month  (n=107) | P value |
| --- | --- | --- | --- | --- |
| Age, years | 63.1±12.1 | 63.1±12.0 | 62.6±13.4 | 0.666 |
| Sex |  |  |  | 0.463 |
| man | 1269(62.5) | 1198(62.3) | 71(66.4) |  |
| women | 760(37.5) | 724(37.7) | 36(33.6) |  |
| Educational level, n (%) |  |  |  | 0.458 |
| elementary or below | 931(45.9) | 888(46.2) | 43(40.2) |  |
| middle school | 411(20.3) | 386(20.1) | 25(23.4) |  |
| high school or above | 687(33.9) | 648(33.7) | 39(36.4) |  |
| Smoking, n (%) |  |  |  | 0.095 |
| Never smoking | 1117(55.1) | 1057(55) | 60(56.1) |  |
| smoking cessation | 403(19.9) | 375(19.5) | 28(26.2) |  |
| current smoking | 509(25.1) | 490(25.5) | 19(17.8) |  |
| Drinking, n (%) | 504(24.8) | 473(24.6) | 31(29) | 0.367 |
| Prior stroke, n (%) | 539(26.6) | 504(26.2) | 35(32.7) | 0.172 |
| NIHSS score on admission, (IQR) | 3.0(1.0,5.0) | 3.0(1.0,5.0) | 4.0(1.0,6.0) | 0.476 |
| Pneumonia during hospitalization, n (%) | 67(3.3) | 67(3.5) | 0(0) | 0.047 |
| BMI, kg/m^2^ | 24.0±3.5 | 24.0±3.5 | 24.0±3.1 | 0.957 |
| SBP on admission (mmHg) | 144.6±20.9 | 144.7±21.0 | 143.7±19.8 | 0.642 |
| DBP on admission (mmHg) | 85.7±12.1 | 85.7±12.2 | 85.7±11.4 | 0.967 |
| Hypertension, n (%) | 1386(68.3) | 1313(68.3) | 73(68.2) | 0.998 |
| Diabetes, n (%) | 435(21.4) | 409(21.3) | 26(24.3) | 0.536 |
| Atrial fibrillation, n (%) | 110(5.4) | 106(5.5) | 4(3.7) | 0.568 |
| **Laboratory findings** |  |  |  |  |
| Total cholesterol (mmol/L) | 4.4±1.0 | 4.4±1.0 | 4.4±0.9 | 0.717 |
| Triglycerides (mmol/L) | 1.7±1.3 | 1.7±1.3 | 1.7±1.6 | 0.902 |
| HDL-C (mmol/L) | 1.1±0.3 | 1.1±0.3 | 1.2±0.4 | 0.060 |
| LDL-Cl (mmol/L) | 2.6±0.8 | 2.6±0.8 | 2.6±0.8 | 0.840 |
| Fast blood glucose (mmol/L) | 5.9±2.3 | 5.9±2.3 | 5.9±2.2 | 0.909 |
| ALT, U/L | 19.0(14.0,28.0) | 19.0(14.0,28.0) | 19.4(13.0,28.5) | 0.919 |
| AST, U/L | 21.0(17.0,27.0) | 21.0(17.0,27.1) | 21.0(17.0,25.0) | 0.622 |
| Alkaline phosphatase (U/L) | 79.1±27.5 | 79.3±27.8 | 76.3±23.0 | 0.283 |
| Homocysteine, μmol/L | 21.5±14.2 | 21.6±14.3 | 19.4±12.2 | 0.225 |
| Serum [creatinine](javascript:;) (mg/L) | 0.8±0.2 | 0.8±0.2 | 0.8±0.1 | 0.436 |
| eGFR, mL/min/1.73 m^2^ | 80.7±10.6 | 80.6±10.6 | 81.7±11.4 | 0.299 |
| Blood Urea Nitrogen, mmol/L | 4.9±1.6 | 4.9±1.6 | 5.1±1.8 | 0.299 |
| Uric Acid, μmol/L | 285.7±91.2 | 285.2±91.1 | 293.5±92.7 | 0.363 |
| Leukocyte values, ×10^9^/L | 6.8±2.4 | 6.9±2.4 | 6.7±2.2 | 0.452 |

Abbreviations: NIHSS, National Institutes of Health Stroke Scale; BMI, body mass index; SBP, systolic blood pressure; DBP, diastolic blood pressure; HDL, high density lipoprotein; LDL, low density lipoprotein; ALT, alanine amino transferase; AST, aspartate amino transferase
